# Serum zinc levels in youth with avoidant/restrictive food intake disorder and anorexia nervosa: Clinical correlation with weight and psychopathology

**DOI:** 10.1177/02601060231191658

**Published:** 2023-07-27

**Authors:** Fauzia Mahr, Marley G Billman Miller, Marlana A Quaill, Sheryl A Ryan, Tania Nadeem

**Affiliations:** 1 Department of Psychiatry and Behavioural Health, 12310 Penn State College of Medicine, Hershey, PA, USA; 2 Department of Pediatrics, 12310 Penn State College of Medicine, Hershey, PA, USA; 3 6242 Department of Psychology, Barry University, Miami, FL, USA; 4 Department of Psychiatry, Aga Khan University, Karachi, Pakistan

**Keywords:** Zinc, anorexia nervosa, avoidant/restrictive food intake disorder, executive functioning, eating disorder

## Abstract

**Background:**

Avoidant/restrictive food intake disorder (ARFID) and anorexia nervosa (AN) are characterized by restrictive eating and micronutrient deficiencies. While zinc deficiency has been identified in AN, zinc level in ARFID has not been systematically assessed.

**Aim:**

Examine serum zinc levels and their association with eating pathology, psychopathology, and executive functioning in youth with ARFID and AN.

**Methods:**

This study included 28 adolescents (*M*_age_ = 13, 75% female) receiving treatment for ARFID (*n* = 13) and AN (*n* = 15). Demographic data and intake mood metrics were obtained via chart review. Participants completed the Delis–Kaplan Executive Functioning Systems and their mothers completed the behavior rating inventory of executive function (BRIEF-2). Zinc level was collected via blood draw. Independent samples *t*-tests, Pearson's chi-square, and Pearson's correlations were used to evaluate between-group differences and the relationship between zinc level and clinical correlates.

**Results:**

No between-groups differences emerged in zinc levels, though half the sample demonstrated low levels for their ages. No significant correlations were found between zinc level and demographic data, mood measures, or executive functioning tasks. AN had relatively lower zinc levels, higher eating pathology, and anxiety, though ARFID had a longer duration of illness. Correlations between zinc and BRIEF-2 scores were mixed.

**Conclusion:**

This is the first study to systematically assess zinc levels in ARFID. While there were no group differences for zinc levels, 50% of the sample had low zinc levels. Zinc level did not correlate with higher psychopathology. Monitoring zinc levels throughout treatment in the context of anabolic processes can inform treatment strategies.

## Introduction

Anorexia nervosa (AN) and avoidant/restrictive food intake disorder (ARFID) are serious eating disorders associated with high morbidity. Both disorders are characterized by restrictive eating patterns and present with micro and macronutrient deficiencies. AN is considered the most lethal psychiatric disorder with high relapse rate and a debilitating course of illness. Individuals with AN primarily reduce their caloric intake secondary to intense fear of weight gain (Hermens et al., 2020). Unlike AN, food restriction in ARFID is not driven by fear of weight gain or body image distortions (Couturier et al., 2019; Norris et al., 2016, Norris et al., 2018). The limited food intake in ARFID stems from a variety of causes including sensory issues, low appetite or lack of interest in food, and fear of aversive consequences of food intake.

Nevertheless, the restricted food consumption in ARFID results in either significant weight loss, dependence on supplemental nutrition, or specific nutritional deficiencies. Malnutrition resulting from food restriction in AN and ARFID severely impacts psychosocial functioning in individuals suffering from these disorders. While many micronutrients have been hypothesized to play a role in the pathophysiology of AN and ARFID, zinc deficiency has been reported in both AN and ARFID patients (Katz et al., 1987; Lucarelli et al., 2017; Nova et al., 2004; Schmidt et al., 2021). Zinc is an essential micronutrient, required for neural and physiological functioning. It is primarily found in the glutamatergic neurons in the brain as well as some other cortical and limbic structures (hippocampus, amygdala; Hermens et al., 2020). Zinc is an allosteric neuromodulator that inhibits NMDA receptors. Zinc deficiency leads to overactivation of these receptors and resultantly produces excitotoxic state by increasing glutamate (Hermens et al., 2020). Low zinc has been associated with sensory impairments, loss of appetite, excessive fatigue, dermatologic abnormalities, mood and behavior abnormalities, slowed learning, and cognitive problems (Greenblatt and Delane, 2018; Hermens et al., 2020).

Cognitive deficits including inflexibility and rigidity have been reported in both ARFID and AN (Grau et al., 2019; Thomas, 2017). Cognitive inflexibility is a critical concept pertaining to eating disorders as it is widely regarded as a predictor for poorer prognosis. Some studies have reported more cognitive impairment with longer duration of illness (Grau et al., 2019) though the data regarding this finding are largely inconclusive. However, the extent to which nutritional status and zinc levels may affect cognitive performance in ARFID is unknown so far necessitating systematic assessment of neuropsychological functioning in this domain.

Additionally, there are no FDA-approved medications for either AN or ARFID, yet a sizeable majority of these individuals receive psychotropic medication despite lack of sound evidence to support their efficacy (Crow, 2019; Flament et al., 2012; Garner et al., 2016; Himmerich and Treasure, 2018). This relative absence of evidence-based psychotropic medication recommendations necessitates consideration of complementary and alternative therapeutic interventions for early intervention and recovery in AN and ARFID.

Zinc supplementation has shown some benefits in AN. Evidence from some randomized, double-blind, controlled clinical trials supports the use of zinc supplementation to improve the rate of weight recovery (Birmingham and Gritzner, 2006). However, another study found no differences in rate of weight gain with zinc supplementation (Lask et al., 1993). The role of zinc in weight restoration in AN therefore remains unclear though zinc insufficiency has been implicated in emotional dysregulation and depression linked to malnutrition (Maes et al., 1994). Zinc deficiency has also been reported in ARFID (Schmidt et al., 2021). However, serum zinc levels in ARFID and their association with eating pathology, recovery, or psychiatric comorbidities have not been systematically assessed.

In view of this gap in literature, we set out to examine the differential effects of nutritional status (as measured by BMI) associated with ARFID and AN, on some elements of proximal brain functioning assessed by serum zinc levels and neuropsychological measures of cognitive and executive functioning in childhood and adolescence.

This cross-sectional study also aimed to examine whether the relationship between variability in nutritional status in ARFID and AN and zinc levels is moderated by nadir BMI, eating pathology, duration of illness, length of stay, and psychiatric comorbidities like depressed mood, anxiety, etc. We hypothesized that zinc levels will be low in both groups and will correlate with BMI, eating pathology, and executive function deficits. We also hypothesized that low serum zinc will be associated with higher psychiatric comorbidities.

## Methods

### Participants

Our sample consisted of 28 patients receiving services for a diagnosis of AN (*n *= 15) or ARFID (*n *= 13). The majority of the sample identified as White/Caucasian (96%, 4% Hispanic/Latinx) and female (75%, 25% male). The mean age of participants was 13 years old, with a slightly older cohort in the AN group (*M *= 14 years old in AN group with range of 10–17 years old, *M *= 12 years old in ARFID group with range of 8–17 years old). More than half (57.0%) of participants were receiving treatment at the partial hospitalization program (PHP), while 43% of participants received outpatient care. Average length of stay in the treatment program was 5.7 weeks, with no significant differences between groups on treatment length.

### Procedures

This cross-sectional study aimed to collect quantitative data that described the characteristics of both groups. Research staff collected informed consent and assent of all participants and their caregivers to participate. Participants agreed for their intake screening assessments, the CDI-II, RCMAS-2, ChEAT, to be used for research purposes. If the participants did not complete the screening assessments at intake, they completed the screening tools after giving consent. Trained research staff administered the two composites on the Delis–Kaplan Executive Functioning Systems (D-KEFS) to the participants. Meanwhile, their caregivers completed the BRIEF-2 rating scale on their child's behavior and executive functioning. Zinc level was collected via blood draw by a certified medical professional within 3 days of the assessments. All zinc samples were analyzed by laboratory staff. This study was approved by the Institutional Review Board of the Penn State College of Medicine (#10658).

### Measures

Zinc and anthropometrics. Zinc level was obtained via blood draw on-site at the hospital's lab by trained medical staff. Height and weight data were collected by nurse staff at the clinic and obtained from electronic medical charts by research staff. BMI and percent of median body weight (%MBW) were calculated by licensed nutritionists that are part of the interdisciplinary care of the clinic.

Children's depression inventory, second edition (CDI-II). The CDI-II was administered as a self-report screening tool to assess for depressive symptoms (Kovacs, 2011). This measure has been validated in youth aged 7–17. There is a clinical cutoff that represents the severity of depressive symptoms, such that total scores of 25 and above indicate severe depression, 20–25 indicate moderate depression, 15–19 indicate mild depression, and 14 and below are not clinically significant. The total score was interpreted in analyses.

Revised children's manifest anxiety scale, second edition (RCMAS-2). The RCMAS-2 was administered as a 49-item self-report screening tool to assess for physiological anxiety, worrying, social concerns, and difficulty with concentration (Reynolds and Richmond, 1985). This measure has been validated in individuals aged 6–19 years old. There is a clinical cutoff that represents the severity of anxiety symptoms, such that total scores of 71 and above indicate clinically significant symptomatology, total scores between 61 and 70 indicate at-risk symptomatology and 60 and below are not clinically significant. The total score was interpreted in analyses.

The children's eating attitude test (ChEAT). The ChEAT was administered as a 26-item self-report tool for eating disorder pathology, such as dieting, restricting, purging, food preoccupation, and oral control (Maloney et al., 1988). This measure was designed for children aged 8–13 years old, but it is routinely utilized at the participating PHP as a marker of change in eating pathology during time in treatment. Thus, this measure was utilized in the present study to capture eating pathology. Participants rated eating disorder symptomatology on a 6-point Likert scale, from never to always. A sum of 20 or more indicates the presence of an eating disturbance. The total score was interpreted in analyses.

D-KEFS. The D-KEFS measures higher level cognitive and executive functions. Executive functioning is related to impulsivity, emotion regulation, and decision-making (Delis et al., 2001). This assessment has been validated in individuals aged 8–89 years old. Research staff administered the verbal fluency composite on the D-KEFS and specifically evaluated performance on the category-switching task. Verbal fluency measures automatic verbal processing, such as the ability to quickly name as many words as possible that fall within a certain category. Category switching measures rapid and accurate mental processing of naming items that belong to a specific category, such as names of furniture or fruits, and switching between categories. The sum of accurate items identified and the total number of switches between categories in this subsection were interpreted in analyses.

Behavior rating inventory of executive functioning-second edition (BRIEF-2). The BRIEF-2 is a rating scale which measures executive functioning in natural environments. Participants’ mothers completed this scale providing data on the participants’ behaviors and executive skills demonstrated within the home (Gioia et al., 2015). This measure has been validated in parents of children aged 5–18 years old. There are nine subscales on the BRIEF-2 that measure different aspects of executive functions: Inhibit (the ability to resist impulses and behaviors at the appropriate time), self-monitor (awareness of how their behavior impacts others), shift (ability to demonstrate flexibility in transitions between environments, activities, or situations), emotional control (ability to regulate emotional reactions), initiate (ability to start an activity without help), working memory (remembering information and applying it to a task), plan (preparedness for a future even by breaking down a task by steps and creating goals), task monitoring (ability to observe details of a task), and organization of materials (ability to maintain order and organization).

Total scores represent clinical significance, such that total scores of 70 and above indicate significant impairment in each corresponding domain, total scores between 65 and 69 indicate potentially elevated impairment, total scores between 60 and 64 indicate mild impairment, and total scores of 59 and below are not clinically significant (little impairment). The global composite (or overall score) and three index scores (BRI: behavioral regulation index, CRI: cognitive regulation index, and ERI: emotional regulation index) were interpreted in analyses, along with each individual subscale score.

### Data analysis

Data were analyzed using IBM SPSS version 28 (IBM, 2021). Independent samples t-tests were used to compare continuous variables, while Pearson's chi-square tests were used to compare categorical variables. According to Hair et al. (2010), Byrne (2010), Curran et al. (1996), as zinc level and all other variables did not violate normality (skewness < 2 and kurtosis < 7), Pearson's correlations were used to assess the relationship between zinc level and other factors, such as age, weight, length of stay, duration of illness, mood measures, and executive functioning.

## Results

### Demographic differences

Table 1 outlines the demographic data of our sample. Notably, participants with ARFID had a significantly longer duration of illness, *t*(1) = −3.05, *p* = .009, while participants with AN scored significantly higher on measures of eating pathology, ChEAT, *t*(1) = 5.06, *p* < .001 and anxiety, RCMAS-II, *t*(1) = 2.13, *p* = .043. No significant differences were found on level of care, length of stay in PHP, BMI, %MBW, or scores on the depression measure (CDI-II).

**Table 1.** Percentages, mean scores, and standard deviations on measures assessing participant demographics.

Variable	AN (*n* = 15)	ARFID (*n* = 13)	Total (*N* = 28)		
	% (*n*)	% (*n*)	% (*n*)	χ^2^	*p*
Gender					
% Female	93.3 (14)	53.8 (7)	75.0 (21)		
% Male	6.7 (1)	46.2 (6)	25.0 (7)	5.79*	.016
Race/ethnicity					
% White/Caucasian	100.0 (15)	92.3 (12)	96.4 (27)		
% Hispanic/Latino	0.0 (0)	7.7 (1)	3.6 (1)	1.20	.274
Level of care					
PHP	73.3 (11)	38.5 (5)	57.1 (16)		
Outpatient clinic	26.7 (4)	61.5 (8)	42.9 (12)	3.46	.063
Zinc					
Low	53.3 (8)	46.2 (6)	50.0 (14)	1.23	.542
Within range	46.7 (7)	46.2 (6)	46.4 (13)		
High	0.0 (0)	7.7 (1)	3.6 (1)		
	*M* (*SD*)	*M* (*SD*)	*M* (*SD*)	*t*	*p*
Age	14.00 (1.77)	12.50 (3.02)	13.30 (2.50)	1.63	.115
Duration of illness (months)	12.67 (11.79)	55.62 (49.58)	32.61 (40.50)	−3.05**	.009
Length of stay in PHP (weeks)	5.46 (3.01)	6.08 (2.77)	5.66 (2.86)	−0.39	.704
BMI	19.36 (1.55)	17.86 (2.72)	18.64 (2.28)	1.77	.089
%MBW	97.41 (6.49)	98.15 (12.70)	95.76 (9.68)	−0.19	.853
Eating pathology					
ChEAT	37.64 (17.06)	12.15 (7.72)	25.37 (18.47)	5.06***	<.001
Anxiety and depression symptoms					
CDI	62.93 (11.14)	54.08 (13.11)	58.67 (12.71)	1.90	.070
RCMAS	57.57 (10.55)	46.69 (15.71)	52.33 (14.15)	2.13*	.043
Zinc level	70.66 (12.24)	82.15 (17.66)	76.00 (15.82)	−2.02	.054

Note: BMI = body mass index, %MBW = % median body weight, ChEAT = Children's Eating Attitudes Test, CDI = Children's Depression Inventory, RCMAS = Revised Children's Manifest Anxiety Scale. *p < .05, **p < .01, ***p < .001.

### Zinc level

Though the between-groups difference in zinc level was not significant, trends suggest that zinc levels for participants with AN are lower than those with ARFID. Rankings of low, within range, and high were derived from previous research (Hennigar et al., 2018). Half of the sample (50.0%) demonstrated abnormally low zinc levels for their ages. One participant with ARFID demonstrated an abnormally high zinc level for their age. Distributions of those with low or within-range zinc levels did not significantly differ by diagnostic group.

### Correlations

Table 2 outlines correlations between zinc level, demographic characteristics, and both parent-reported and objectively measured neuropsychological functioning. No significant correlations were found between zinc level and age, BMI, %MBW, duration of illness, or length of stay in PHP. No significant correlations were found between zinc level and scores on the ChEAT, RCMAS-II, or CDI-II. No significant correlations were found between zinc level and participant performance on the verbal fluency task of the D-KEFS.

**Table 2.** Pearson *r* values of correlates with age, weight, length of stay, duration of illness, mood measures, and executive functioning.

Variable	Inhibit	Self-monitor	Shift	Emotional control	Initiate	Working memory	Plan	Task monitoring	Organization of materials	Behavioral regulation index	Cognitive regulation index	Emotion regulation index	Global executive composite
Zinc level	−0.30	−0.46*	−0.37	−0.45*	−0.36	−0.31	−0.30	−0.30	−0.28	−0.41	−0.28	0.57**	0.59**
Variable	Age	BMI	%MBW	LOS	DOI	CDI	RCMAS	ChEAT	D-KEFS category switching total	D-KEFS verbal fluency accuracy			
Zinc level	0.01	−0.27	−0.30	0.22	0.26	0.19	0.19	−0.09	−0.17	−0.05			

Note: BMI = body mass index; %MBW = % median body weight; LOS = length of stay in weeks; DOI = duration of illness in months; CDI = children's depression inventory; RCMAS = revised child manifest anxiety scale; ChEAT = children's eating attitudes test; BRIEF = behavior rating inventory of executive functioning. Higher score = greater dysfunction. *p < .05, **p < .01, ***p < .001.

**Figure 1.**
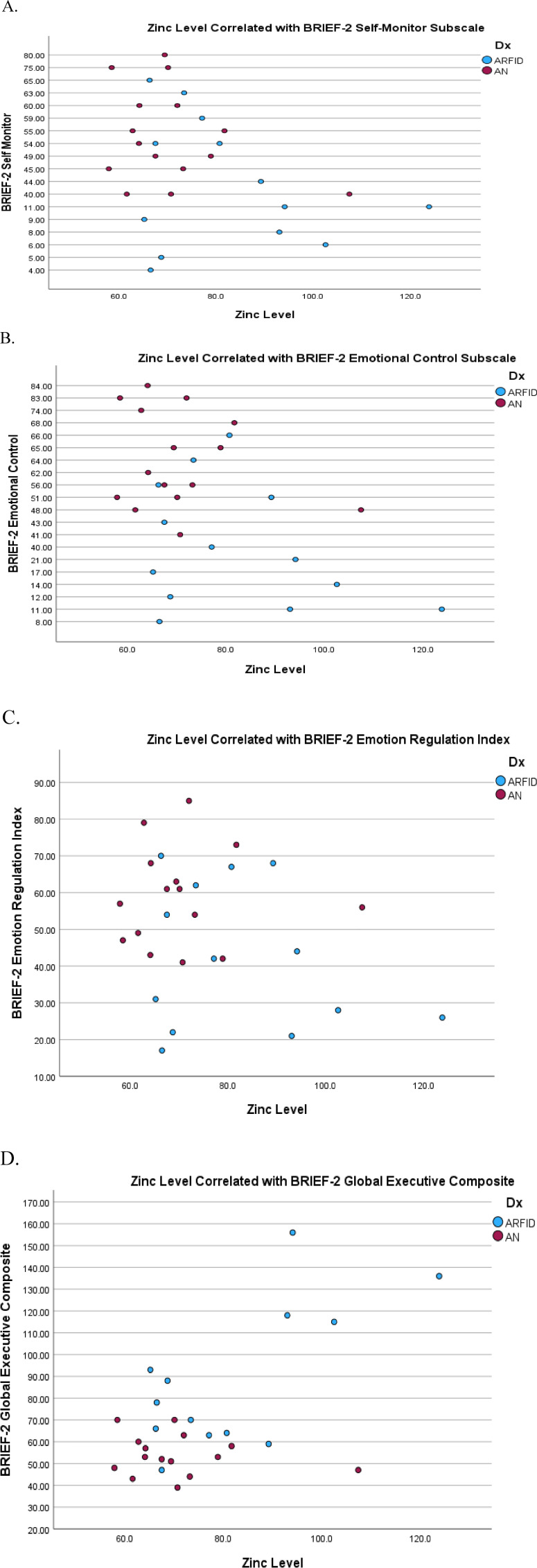
Scatter plots of significant correlations with serum zinc level.

On the parent-reported measure of the participant's neuropsychological functioning (BRIEF-2), several significant correlations were found. Significant negative correlations were found on the self-monitor, *r*(27) = −0.46, *p *= .015 and emotional control, *r*(27) = −0.45, *p *= .016 subscales, indicating that lower zinc level is associated with greater dysfunction in these areas. Interestingly, significant positive correlations were found on the cognitive regulation index, *r*(27) = 0.57, *p *= .001 and the global executive composite, *r*(27) = 0.59, *p *= .001, indicating that lower zinc levels are associated with lower rates of dysfunction overall and cognitive regulation. Significant correlations are depicted in Figure 1.

## Discussion

This is the first study that has systematically compared zinc levels in ARFID and AN participants. Zinc level was low in half of the participants in both groups. Zinc level for AN participants was lower than in ARFID and AN patients had significantly higher scores on measures of eating pathology and anxiety. Zinc levels hold interest in restrictive disorders because some researchers have noted their effects on taste and appetite stimulation (Naureen et al., 2014). Zinc levels are, however, highly sensitive to catabolic and anabolic states and levels can rapidly shift with changes in metabolism and energy requirements of the body (Golden and Golden, 1981; Nova et al., 2004a). Additionally, despite restrictive intake, serum zinc levels are generally maintained in AN. Our results confirm some of these findings; half of the participants had low zinc levels during the course of treatment. It is noteworthy that no significant correlations were found between the length of treatment and level of care (partial hospitalization vs. outpatient care).

We also did not find any significant correlations between serum zinc level and BMI; thus, our primary hypothesis that zinc levels will correlate with BMI, was not supported. There were no significant correlations between zinc levels, age, %MBW, or duration of illness. ARFID participants had significantly longer duration of illness but their %MBW was not statistically different than AN participants though their zinc levels were relatively higher than AN participants.

Lieberman et al. (2019) reported that ARFID participants experienced higher learning difficulties and neurodevelopmental disorders. We measured higher level cognitive and executive functions in participants using D-KEFS and obtained mother's report of executive functioning in natural environment using BRIEF-2. The results from the participants and mothers did not match. These results are consistent with some previous studies which report disagreement in parent and child reports of depression and anxiety in offspring (Baumgartner et al., 2020; Faraone et al., 1995; Tompson et al., 2010). Mothers reported decreased emotional control and self-monitoring which correlated with lower levels of zinc while the participant results indicated higher emotional regulation and high global executive composite score with low zinc levels. It is likely that maternal reports were impacted by the child's distress and maternal response to their challenges with eating, other psychopathology and functional impairment that led to requiring medical intervention. Additionally, maternal psychopathology could have contributed to these reports as noted in the literature previously (Kroes et al., 2003; Tompson et al., 2010).

It is also likely that the lower zinc levels were secondary to anabolic demands during nutritional recovery and therefore represented a recovery phase. Literature does support decrease in zinc levels with increase in anabolic processes due to greater need for nutrients during the process of nutritional rehabilitation (Golden and Golden, 1981; Nova et al., 2004a). However, definitive conclusions regarding low zinc levels and participant reports of higher emotional regulation cannot be drawn because of the cross-sectional nature of this study. This finding certainly necessitates further exploration of zinc levels during various treatment phases to identify factors that may favor better treatment outcomes.

Nevertheless, there are some study limitations that merit acknowledgment. First, this was a cross sectional study, so definitive causal conclusions cannot be drawn, nor does it represent changes across the spectrum of treatment. Second, we tested participants with two different diagnosis, though with some known overlapping characteristics. The participants were in different levels of care (outpatient vs. PHP), however. there were no significant associations with level of care which could impact the results. Third, serum zinc level does not always reflect the body store of zinc and its level is affected by the anabolic or catabolic phases. Also, all of our participants were receiving multivitamins secondary to being in treatment settings, which may impact the zinc levels. Additionally, there was a greater number of female participants compared to males, therefore conclusions about gender comparisons cannot be accurately drawn. Lastly, the parent-reported measures are not objective measurements. We failed to measure maternal psychopathology which may have impacted maternal reports of their offspring's emotional regulation status.

## Conclusion

This is the first study to compare zinc levels in ARFID and AN and their correlation with measures of psychopathology and neuropsychological functioning. Despite its limitations, this cross-sectional study provides meaningful pilot conclusions that BMI and zinc levels are not correlated and low zinc does not correlate with higher psychopathology in ARFID. AN participants had lower zinc levels and higher eating disorder pathology compared to ARFID. An unexpected finding was higher cognitive regulation with lower zinc levels which suggests that monitoring zinc levels at different stages in treatment process and assessing zinc levels in the context of anabolic processes during nutritional and weight recovery might be beneficial to identify its role in treatment outcomes. The variability in zinc levels between ARFID and AN suggests that further research focusing on disorder-specific nutritional deficiencies and systematic neuropsychological assessments during various phases of treatment and nutritional rehabilitation is essential.
